# Resveratrol Modulates SIRT1 and DNMT Functions and Restores LINE-1 Methylation Levels in ARPE-19 Cells under Oxidative Stress and Inflammation

**DOI:** 10.3390/ijms19072118

**Published:** 2018-07-20

**Authors:** Andrea Maugeri, Martina Barchitta, Maria Grazia Mazzone, Francesco Giuliano, Guido Basile, Antonella Agodi

**Affiliations:** 1 Department of Medical and Surgical Sciences and Advanced Technologies “GF Ingrassia”, University of Catania, via S. Sofia, 87, 95123 Catania, Italy; andreamaugeri88@gmail.com (A.M.); martina.barchitta@unict.it (M.B.); 2 SIFI SpA, Research and Development Department, Via Ercole Patti 36, 95025 Catania, Italy; mariagrazia.mazzone@sifigroup.com (M.G.M.); francesco.giuliano@sifigroup.com (F.G.); 3 Department of General Surgery and Medical-Surgical Specialties, University of Catania, Via Plebiscito, 628, 95124 Catania, Italy; gbasile@unict.it

**Keywords:** retinal degeneration, DNA methylation, epigenetics, oxidative stress, inflammation

## Abstract

The role of epigenetic alterations in the pathogenesis of retinal degenerative diseases, including age-related macular degeneration (AMD), has been pending so far. Our study investigated the effect of oxidative stress and inflammation on DNA methyltransferases (DNMTs) and Sirtuin 1 (SIRT1) functions, as well as on long interspersed nuclear element-1 (LINE-1) methylation, in human retinal pigment epithelial (ARPE-19) cells. Therefore, we evaluated whether treatment with resveratrol may modulate DNMT and SIRT1 functions and restore changes in LINE-1 methylation. Cells were treated with 25 mU/mL glucose oxidase (GOx) or 10 µg/mL lipopolysaccharide (LPS) to mimic oxidative or inflammatory conditions, respectively. Oxidative stress decreased DNMT1, DNMT3a, DNMT3b, and SIRT1 expression (*p*-values < 0.05), as well as total DNMTs (−28.5%; *p* < 0.0001) and SIRT1 (−29.0%; *p* < 0.0001) activities. Similarly, inflammatory condition decreased DNMT1 and SIRT1 expression (*p*-values < 0.05), as well as total DNMTs (−14.9%; *p* = 0.007) and SIRT1 (−20.1%; *p* < 0.002) activities. Interestingly, GOx- and LPS-treated cells exhibited lower LINE-1 methylation compared to controls (*p*-values < 0.001). We also demonstrated that treatment with 10 μM resveratrol for 24 h counteracted the detrimental effect on DNMT and SIRT1 functions, and LINE-1 methylation, in cells under oxidative and inflammatory conditions. However, further studies should explore the perspectives of resveratrol as a suitable strategy for the prevention and/or treatment of retinal degenerative diseases.

## 1. Introduction

Oxidative stress and inflammation are shared pathological features across retinal degenerative diseases. Among these, age-related macular degeneration (AMD) is the most common cause of blindness in developed countries, with a prevalence that ranges from 2% to 20% among elderly people [1]. Overall, the pathological process of AMD leads to the progressive destruction of the neurosensory macular area, involving retinal pigment epithelium (RPE), Bruch’s membrane, and choroid [2]. The early stages of AMD are characterized by the aberrant pigmentation of the RPE and the accumulation of extracellular deposits of lipid, cellular debris, and proteins (i.e., drusen). The advanced stages may manifest as nonexudative or exudative AMD: the first is characterized by the geographic atrophy of RPE and thinning of the retina; the second is characterized by the development of choroidal neovascularization (CNV), which negatively affects central vision [3,4]. AMD is one of the most investigated multifactorial diseases since several sociodemographic (age and race) [5], environmental (cigarette smoking, light exposure, and nutrient intake) [6,7,8,9,10], and genetic risk factors [11,12,13,14] can act together, leading to a chronic condition of oxidative stress and inflammation [15].

Hydrogen peroxide (H_2_O_2_) treatment is one of the most commonly used models to test oxidative stress susceptibility in vitro and oxidative damage in vivo. However, the effect of H_2_O_2_ is dose-dependent and ranges from cell proliferation, migration, survival, and differentiation [16,17] to cell death [18,19]. Exposure to glucose oxidase (GOx), which maintains stable H_2_O_2_ concentration, is alternatively used. In vivo studies also demonstrated that ocular injection of lipopolysaccharide (LPS) in rats stimulates the synthesis and release of proinflammatory mediators, such as nitric oxide, platelet-activating factor, tumor necrosis factor alpha (TNFα), interleukin 1 beta (IL1β), and other cytokines [20,21]. In line with these findings, human retinal pigment epithelial cells (ARPE-19) treated with LPS exhibited increased levels of IL-6 and IL-8 [22,23,24], as well as nuclear factor-kappa B (NF-κB) translocation from the cytoplasm to the nucleus [22]. Interestingly, retinal cells show altered gene expression in response to exogenous and endogenous stimuli [25], suggesting a typical gene–environment interaction [26]. In this context, epigenetic mechanisms, especially DNA methylation and histone modifications, might modulate the interaction between genetic factors and environmental exposures [27], affecting both gene expression and genome stability [28,29]. However, the significance of epigenetic alterations in the pathogenesis of retinal degenerative diseases in general, and AMD in particular, has been pending so far.

The methylation process is carried out by DNA methyltransferases (DNMTs), out of which only DNMT1, DNMT3A, and DNMT3B are catalytically active [30]. In mammals, the methylation process occurs at short DNA sequences (i.e., CpG islands), which typically contain around 5–10 CpGs per 100 bp. Up to 80% of CpG islands are localized in noncoding regions scattered throughout the genome (e.g., satellite repeat, short interspersed nuclear elements, and long interspersed nuclear element-1(LINE-1)) that mainly contribute to the global methylation status [30]. LINE-1 sequences, accounting for ≈18% of the human genome, are widely used as a surrogate marker of global methylation in aging and age-related diseases [31,32,33,34].

Sirtuin 1 (SIRT1), one of the seven mammalian homologs (SIRT1–SIRT7) of yeast silent information regulator 2, is a NAD+-dependent histone deacetylase with multiple roles in aging, apoptosis, DNA repair, inflammation, and oxidative stress [35]. Although DNA methylation and histone deacetylation are distinct biochemical processes that control gene expression, SIRT1 regulates the activities of DNMT1, the enzyme responsible for maintenance of DNA methylation [36].

Resveratrol (2,3,4′-trihydroxystilbene), a flavonoid associated with the cardiovascular benefits of red grapes and wine, has been shown to significantly increase SIRT1 activity through allosteric interaction, increasing SIRT1 affinity for both NAD+ and the acetylated substrate [37,38]. More recently, due to its antioxidant, anti-inflammatory, and antiangiogenic properties, resveratrol has been also proposed as a candidate for the treatment of ocular diseases [39].

The present study investigated the effect of oxidative stress and inflammation on retinal DNMT and SIRT1 functions, as well as on LINE-1 methylation levels, in ARPE-19 cells. Therefore, we evaluated whether treatment with resveratrol may modulate DNMT and SIRT1 functions and restore changes in LINE-1 methylation.

## 2. Results

### 2.1. Oxidative Stress and Inflammatory Conditions Affect Cell Viability in ARPE-19 Cells

**Figure 1:**
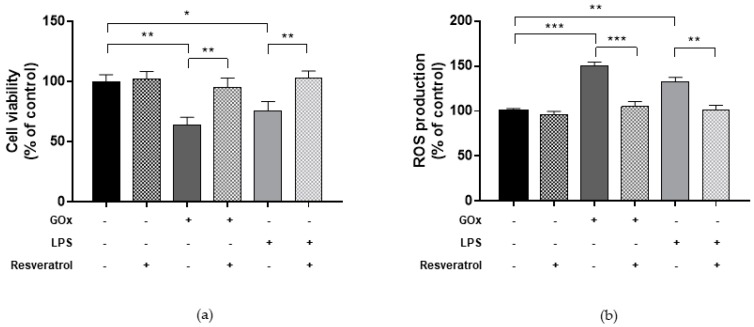
Cell viability and reactive oxygen species (ROS) production in human retinal pigment epithelial (ARPE-19) cells under oxidative stress and inflammatory conditions. (**a**) Thiazolyl blue tetrazolium bromide (MTT) assay showed that treatment with 25 mU/mL glucose oxidase (GOx) or 10 µg/mL lipopolysaccharide (LPS) for 24 h reduced cell viability by 35.8% (*p* = 0.004) and 24.2% (*p* = 0.035), respectively. (**b**) The determination of ROS using 2′,7′-dichlorofluorescin diacetate (DCFDA) demonstrated higher ROS production in GOx- and LPS-treated cells compared to controls (50.1%, *p* < 0.001; 32.6%, *p* = 0.004; respectively). Resveratrol restores (**a**) viability and (**b**) ROS production in cells under oxidative and inflammatory conditions. The experiments were performed in triplicate and repeated three times. Bar graphs show mean ± SE. * *p* < 0.05, ** *p* < 0.01, *** *p* < 0.001 based on the Student’s *t*-test.

ARPE-19 cells were treated with GOx to mimic a condition of oxidative stress through the continuous production of H_2_O_2_, which leads to reactive oxygen species (ROS) production and a cytotoxic effect of less than 50% [40]. Consistent with a previous study [40] treatment with 25 mU/mL GOx for 24 h reduced cell viability by 35.8% (*p* = 0.004) and increased ROS production by 50.1% compared to untreated cells (*p* < 0.001) (Figure 1).

Similarly, ARPE-19 cells were treated with LPS (type *Escherichia coli*, serotype 0127:B8) to mimic an inflammatory condition [23], which leads to a cytotoxic effect of less than 50%. Consistent with a previous study [22], treatment with 10 µg/mL LPS for 24 h reduced cell viability by 24.2% (*p* = 0.035). Interestingly, treatment with 10 µg/mL LPS for 24 h significantly increased ROS production by 32.6% compared to untreated cells (*p* = 0.004) (Figure 1). According to these results, treatments of ARPE-19 with 25 mU/mL GOx or 10 µg/mL LPS for 24 h were applied for further experiments.

### 2.2. Oxidative Stress Affects DNMT and SIRT1 Functions and LINE-1 Methylation in ARPE-19

**Figure 2:**
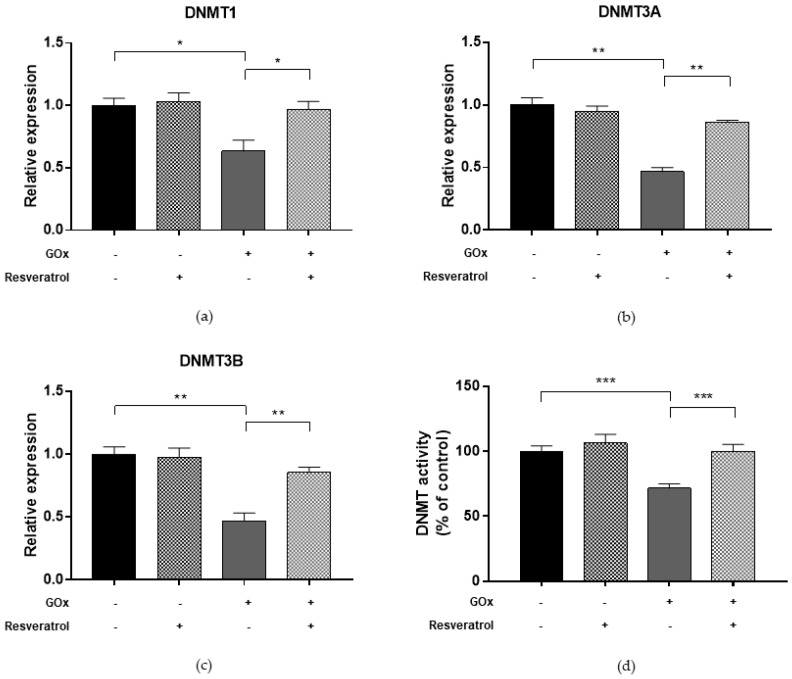
DNA methyltransferase (DNMT) expression and activity in ARPE-19 cells under oxidative stress. (**a**–**c**) Analysis of gene expression showed that treatment with 25 mU/mL GOx for 24 h downregulated DNMT1, DNMT3A, and DNMT3b expression levels (FC = 0.63, FC = 0.47, and FC = 0.46, respectively; *p*-values < 0.05). (**d**) Analysis of total DNMT enzymatic activity using a colorimetric assay confirmed that total DNMT activity was reduced by 28.5% in GOx-treated cells compared to controls (*p* < 0.0001). Treatment with 10 μM resveratrol for 24 h restores DNMT expression (FC = 0.97, *p* = 0.039 for DNMT1; FC = 0.86, *p* = 0.005 for DNMT3A; FC = 0.85, *p* = 0.008 for DNMT3B) and activity (99.9%, *p* = 0.001) in cells under oxidative stress. The experiments were performed in triplicate and repeated three times. Bar graphs show mean ± SE. * *p* < 0.05, ** *p* < 0.01, *** *p* < 0.001 based on the Student’s *t*-test.

**Figure 3:**
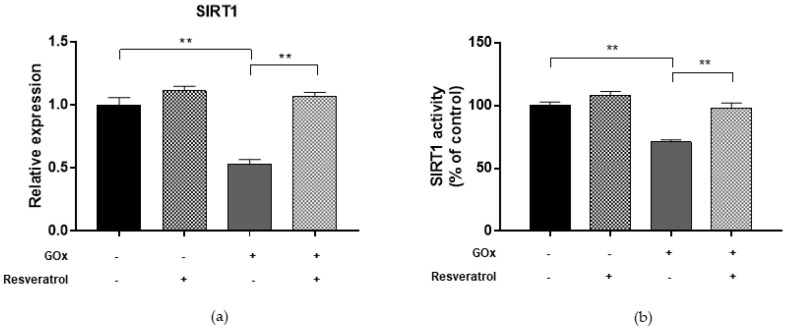
SIRT1 expression and activity in ARPE-19 cells under oxidative stress. (**a**) Analysis of gene expression showed that treatment with 25 mU/mL GOx for 24 h downregulated SIRT1 expression level (FC = 0.53; *p* = 0.002). (**b**) Analysis of SIRT1 enzymatic activity using a fluorimetric assay confirmed that total SIRT1 activity was reduced by 29.0% in GOx-treated cells compared to controls (*p* < 0.0001). Treatment with 10 μM resveratrol for 24 h restores SIRT1 expression (FC = 1.07, *p* = 0.003) and activity (98.0%, *p* = 0.004) in cells under oxidative stress. The experiments were performed in triplicate and repeated three times. Bar graphs show mean ± SE. ** *p* < 0.01, based on the Student’s *t*-test.

**Figure 4:**
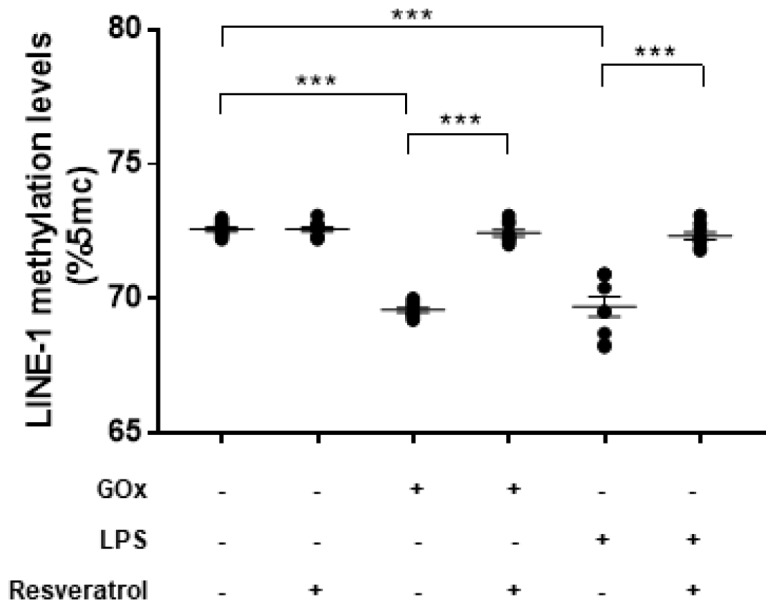
LINE-1 methylation levels in ARPE-19 cells under oxidative stress and inflammatory conditions. Compared to controls (72.6%5mc ± 0.1), LINE-1 methylation levels were lower in GOx- (69.6%5mc ± 0.1; *p* < 0.0001)- and LPS- (69.7%5mc ± 0.4; *p* < 0.0001) treated cells. Treatment with 10 μM resveratrol for 24 h restores LINE-1 methylation levels in cells under oxidative stress (72.4%5mc ± 0.1; *p* > 0.05) and inflammatory (72.3%5mc ± 0.1; *p* > 0.05) conditions. The experiments were performed in triplicate and repeated three times. *** *p* < 0.001 based on the Student’s *t*-test.

To determine whether oxidative stress may affect the DNA methylation process, we first evaluated DNMT functions in ARPE-19 cells treated with 25 mU/mL GOx for 24 h (Figure 2). Compared to untreated cells, GOx treatment decreased DNMT1, DNMT3a, and DNMT3b expression levels (FC = 0.63, FC = 0.47, and FC = 0.46, respectively; *p*-values < 0.05). In addition, total DNMT activity was reduced by 28.5% in GOx-treated cells compared to untreated cells (*p* < 0.0001). Since DNMT functions, especially DNMT1, are regulated by SIRT1 [27], we hypothesized that GOx treatment might also affect SIRT1 expression and activity. Interestingly, we demonstrated that GOx treatment decreased SIRT1 expression (FC = 0.53; *p* = 0.002) and activity (−29.0%; *p* < 0.0001) compared to untreated cells (Figure 3). To evaluate the effect on global DNA methylation, we measured methylation levels of LINE-1, a surrogate marker of global DNA methylation. In line with reduced DNMTs and SIRT1 functions, LINE-1 methylation levels were lower in GOx-treated cells compared to untreated ones (69.6%5mc ± 0.1 vs. 72.6%5mc ± 0.1; *p* < 0.0001) (Figure 4).

### 2.3. Inflammatory Condition Affects DNMTs and SIRT1 Functions and LINE-1 Methylation in ARPE-19

**Figure 5:**
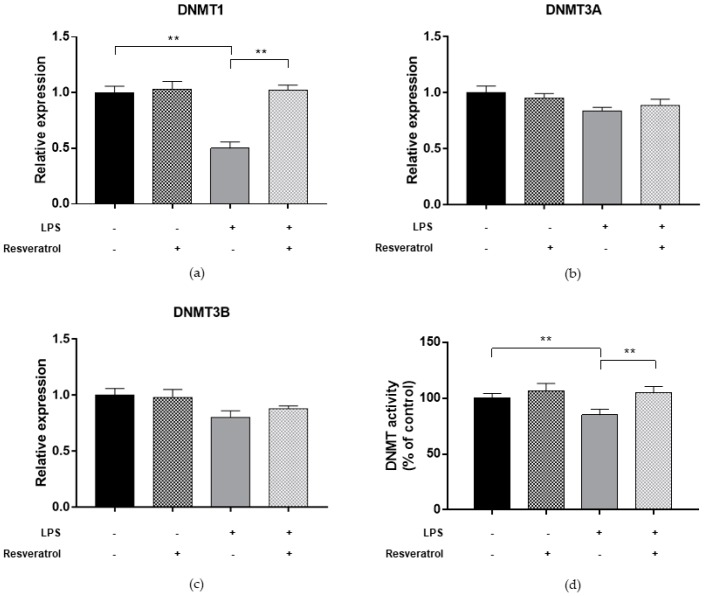
DNMT expression and activity in ARPE-19 cells under inflammatory condition. (**a**) Analysis of gene expression showed that treatment with 10 µg/mL LPS for 24 h downregulated DNMT1 expression level (FC = 0.50; *p* = 0.004), while (**b**,**c**) DNMT3A and DNMT3B expression seemed to be unaffected. (**d**) Analysis of total DNMT enzymatic activity using a colorimetric assay confirmed that total DNMT activity was reduced by 14.9% in LPS-treated cells compared to controls (*p* = 0.007). Treatment with 10 μM resveratrol for 24 h restores DNMT1 expression (FC = 1.02, *p* = 0.002) and total DNMTs activity (104.8%, *p* = 0.009) in cells under inflammatory condition. The experiments were performed in triplicate and repeated three times. Bar graphs show mean ± SE. ** *p* < 0.01 based on the Student’s *t*-test.

**Figure 6:**
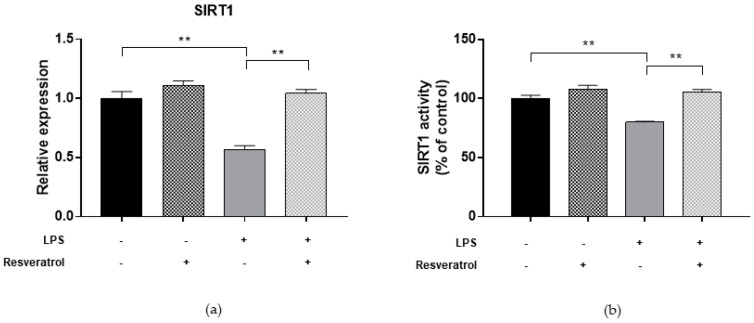
SIRT1 expression and activity in ARPE-19 cells under inflammatory condition. (**a**) Analysis of gene expression showed that treatment with 10 µg/mL LPS for 24 h downregulated SIRT1 expression level (FC = 0.57; *p* = 0.003). (**b**) Analysis of SIRT1 enzymatic activity using a fluorimetric assay confirmed that total SIRT1 activity was reduced by 20.1% in LPS-treated cells compared to controls (*p* = 0.002). Treatment with 10 μM resveratrol for 24 h restores SIRT1 expression (FC = 1.04, *p* = 0.009) and activity (105.5%, *p* = 0.005) in cells under inflammatory condition. The experiments were performed in triplicate and repeated three times. Bar graphs show mean ± SE. ** *p* < 0.01 based on the Student’s *t*-test.

To determine whether inflammatory condition may affect the DNA methylation process, we first evaluated DNMT functions in ARPE-19 cells treated with 10 µg/mL LPS for 24 h (Figure 5). Previous studies reported that treatment of RPE cells with LPS increased the expression of proinflammatory cytokines IL-6 and IL-8 [22,23]. Our study added to the current knowledge, demonstrating that LPS-treated cells exhibited lower DNMT1 expression level (FC = 0.50; *p* = 0.004), while DNMT3A and DNMT3B expression seemed to be unaffected. In addition, treatment with LPS reduced total DNMT activity by 14.9% (*p* = 0.007). Compared to untreated cells, we also showed that LPS treatment decreased both SIRT1 expression (FC = 0.57; *p* = 0.003) and activity (−20.1%; *p* = 0.002) (Figure 6). In line with these results, treated cells exhibited lower LINE-1 methylation levels compared to untreated ones (69.7%5mc ± 0.4 vs. 72.6%5mc ± 0.1; *p* < 0.0001) (Figure 4).

### 2.4. Resveratrol Ameliorates Viability and ROS Production in Cells under Oxidative and Inflammatory Conditions

We also aimed to demonstrate the antioxidant and anti-inflammatory effect of resveratrol against GOx- and LPS-induced changes in ARPE-19 cells. First, we determined viability of cells exposed to various concentrations of resveratrol (1–10 μM) for 24 h. In line with a previous study [41], we found that treatment with 1–10 μM resveratrol for 24 h did not affect viability of ARPE-19 cells. Similarly, resveratrol treatment (1–10 μM) of control cells did not induce changes in ROS production (data not shown). However, Figure 1 shows that treatment with 10 μM resveratrol for 24 h was able to ameliorate cell viability and to alleviate ROS production in ARPE-19 cells under oxidative stress and inflammatory conditions (*p*-values < 0.05 vs. GOx- or LPS-treated cells).

### 2.5. Resveratrol Restores DNMT and SIRT1 Functions and LINE-1 Methylation in Cells under Oxidative and Inflammatory Conditions

Finally, we evaluated whether resveratrol may modulate DNMTs and SIRT1 functions and restore changes in LINE-1 methylation. We demonstrated that treatment with 10 μM resveratrol for 24 h restored both the expression (FC = 0.97, *p* = 0.039 for DNMT1; FC = 0.86, *p* = 0.005 for DNMT3A; FC = 0.85, *p* = 0.008 for DNMT3B; FC = 1.07, *p* = 0.003 for SIRT1) and activity (99.9%, *p* = 0.001 for DNMTs and 98.0%, *p* = 0.004 for SIRT1) of DNMTs (Figure 2) and SIRT1 (Figure 3) in ARPE-19 cells under oxidative stress conditions. Similarly, resveratrol increased DNMT1 expression (FC = 1.02, *p* = 0.002) and total DNMTs activity (104.8%, *p* = 0.009) (Figure 5), as well as SIRT1 expression (FC = 1.04, *p* = 0.009) and activity (105.5%, *p* = 0.005) (Figure 6) in cells under inflammatory conditions. In line with these results, resveratrol also restored LINE-1 methylation levels in cells under oxidative stress (72.4%5mc ± 0.1; *p* < 0.001) and inflammatory (72.3%5mc ± 0.1; *p* < 0.001) conditions (Figure 4).

## 3. Discussion

Efforts to understand the mechanisms underpinning the multifactorial nature of retinal degenerative diseases have led us to explore the DNA methylation process in RPE cells under oxidative stress and inflammation, which interplay in retinal degeneration [42]. In support of this evidence, we observed that RPE cells treated with LPS to mimic an inflammatory condition with increased expression of proinflammatory cytokines [22,23] exhibited increased ROS production compared to untreated cells. In fact, a chronic low-level inflammation status might be exacerbated over time by the accumulation of oxidation products, which, in turn, cause tissue damage and impairment of central vision [36].

To our knowledge, the present work demonstrated for the first time that oxidative stress and inflammatory conditions reduced DNMT and SIRT1 functions, as well as LINE-1 methylation levels, in RPE cells. The slight but significant difference (≈3%) in LINE-1 methylation levels between treated and untreated cells could be partially due to reduced DNMT function. Decreased LINE-1 methylation leads to genomic instability and plays a crucial role in the development of chronic degenerative diseases [31,32,33,34]. However, further research should assess to what extent LINE-1 hypomethylation contributes to retinal degeneration. Although the effect of oxidative and inflammatory conditions on retinal LINE-1 methylation was similar, inflammation seemed to affect only DNMT1 expression, that is, the maintenance DNMT, while oxidative stress also downregulated de novo DNMT3a and DNMT3b, which in turn enable key epigenetic modifications for cellular differentiation and transcriptional regulation [43]. By contrast, a recent study on a model of diabetic retinopathy in RPE cells demonstrated that hyperglycemia-induced oxidative stress upregulates DNMT functions with no effect on LINE-1 methylation, suggesting that retinal degenerative diseases might display distinct DNA methylation profiles [44].

Several lines of evidence proposed the relationship of oxidative stress and inflammation with DNA methylation in retinal degenerative diseases, especially in AMD. A previous study, comparing DNA methylation between AMD patients and age-matched controls, revealed that glutathione S-transferase isoforms mu1 (GSTM1) and mu5 (GSTM5) undergo epigenetic repression in AMD RPE/choroid via promoter hypermethylation, which, in turn, decreased mRNA and protein levels [45]. These enzymes play an important role in the detoxification of electrophilic compounds, including products of oxidative stress, by conjugation with glutathione. Reduced activity of GSTM1 and GSTM5 could affect protection from genome-damaging oxidants with increased vulnerability to oxidative insults. Wei et al. investigated genome-wide differences in DNA methylation between three pairs of twins (both monozygotic and dizygotic) with discordant AMD [46]. Their results, further validated in discordant siblings for AMD and in an AMD case-control cohort, reported significantly decreased interleukin 17 receptor C (IL17RC) promoter methylation in AMD patients, which, in turn, led to increased expression of its protein and mRNA in peripheral blood and in the retina [46]. The *IL17RC* gene encodes for an essential subunit of the IL-17 receptor complex that modulates activity of proinflammatory IL-17A and IL-17F. Although these findings were not confirmed in a subsequent study [47], the putative epigenetic mechanism by which proinflammatory stimuli could promote AMD pathology should be investigated.

Recently, several lines of evidence suggested that SIRT1, a NAD+-dependent histone deacetylase, protects RPE cells against apoptosis and counteracts changes in RPE functions induced by oxidative stress and chronic inflammation. In fact, SIRT1 might be involved in retinal degeneration via modulating cell senescence, DNA damage repair, and apoptosis [35]. Previous studies demonstrated that SIRT1 expression significantly decreased with increasing age in retinal stem cells, and that it was downregulated in human AMD retinas compared to non-AMD donors [48]. Consistently, we reported for the first time that RPE cells under oxidative and inflammatory conditions exhibited decreased SIRT1 expression and activity compared to untreated cells. Since SIRT1 regulates the activities of DNMT, especially DNMT1 [36], these results might explain how oxidative stress and inflammation affect the DNA methylation mechanisms. Interestingly, SIRT1 also attenuated changes induced by Amyloid beta (Aβ), a known constituent of drusen, which induces chronic inflammation [49]. In fact, treatment with a SIRT1 agonist (i.e., SRT1720) restored Aβ-induced upregulation of IL-6, IL-8, and matrix metalloproteinase-9 (MMP-9); this inhibitory effect was abolished in SIRT1 knockdown cells [49]. In addition, a mutual effect between SIRT1 and NF-κB has been established: if, on one hand, SIRT1 inhibits the activation of an NF-κB-mediated inflammatory pathway, on the other hand, NF-κB signaling and inflammatory response can suppress SIRT1 activity [50]. A previous study demonstrated that SIRT1 inhibition by nicotinamide decreased the secretion of proangiogenic factors in ARPE-19 cells, suggesting that treatment with SIRT1 inhibitors might be a potential strategy against angiogenesis [51]. By contrast, others revealed that treatment with resveratrol, a natural SIRT1 activator, restored VEGF secretion in RPE cells under oxidative and inflammatory conditions [52]. Interestingly, resveratrol also downregulated VEGF expression in choroidal endothelial cells by modulating the SIRT1 pathway [53]. In fact, resveratrol stimulates SIRT1 activity through allosteric interaction and increases SIRT1 affinity for both NAD+ and the acetylated substrate [37,38]. Thus, further studies should be encouraged to elucidate the role of SIRT1 in the development of CNV.

Beyond the antioxidant and anti-inflammatory properties, recent literature reported the antiaging effect of resveratrol in epithelial, endothelial, and corneal wound healing [54,55,56]. In the present study, we confirmed that treatment with 10 μM resveratrol for 24 h ameliorated cell viability and ROS production in ARPE-19 cells under oxidative and inflammatory stress. Moreover, we demonstrated that it also counteracted the detrimental effect on DNMT and SIRT1 functions, and LINE-1 methylation. However, further research should investigate whether the protective effect of resveratrol relies on SIRT1 activation rather than on its antioxidant and anti-inflammatory properties.

## 4. Materials and Methods

### 4.1. Cell Culture and Treatments

ARPE-19, purchased from the American Type Culture Collection (Manassas, VA, USA), were maintained in Dulbecco’s Modified Eagle’s medium (DMEM) supplemented with 10% fetal bovine serum (Gibco BRL, Grand Island, NY, USA), 100 U⁄mL of penicillin, and 100 µg⁄mL of streptomycin (Gibco BRL). Cells between 6–10 passages were used in all experiments and incubated at 37 °C and 5% CO_2_. Medium was changed every 48 h.

To mimic conditions of oxidative stress and inflammation, cells reaching 80–90% of confluence were starved in serum-free DMEM and treated with 25 mU/mL GOx or 10 µg/mL LPS (type *Escherichia coli*, serotype 0127:B8; Sigma Chemical, Missouri, MO, USA) for 24 h, respectively. The concentrations of GOx and LPS were chosen according to previously published studies that used the same cell line [22,40]. To investigate whether resveratrol might restore changes induced by GOx and LPS, during treatments cells were also incubated with 10 μM resveratrol for 24 h. This concentration was chosen according to results of cell viability after incubation with increasing concentrations (1–10 μM) of resveratrol for 24 h. All experiments were performed in triplicate for three times on different days.

### 4.2. Determination of Cell Viability

After treatment with GOx, LPS, and resveratrol, alone or in combination, we evaluated cell viability by using the Thiazolyl blue tetrazolium bromide (MTT) assay. Treated and untreated cells were briefly seeded at a density of 2.0 × 104 cells/well in a 96-well plate and incubated for 24 h. Then, cells were incubated with MTT (1.6 mg/mL) at 37 °C for 4 h. After removing the solution, cells were resuspended in 100 µL of dimethyl sulfoxide and optical density was read at 540 with an optional reference wavelength of 670 nm. Cell viability was reported as percentage of control.

### 4.3. Determination of ROS

Intracellular ROS levels were determined using the Abcam Cellular ROS Detection Assay Kit according to manufacturer’s instructions (Abcam plc, Cambridge, UK). In brief, cells were seeded at a density of 2.0 × 104 cells/well in a dark, clear bottom 96-well microplate. Cells were rinsed with 100 μL/well of 1× Buffer and stained by adding 100 μL/well of the redox-sensitive fluoroprobe 2′,7′–dichlorofluorescin diacetate (DCFDA) for 45 min at 37 °C in the dark. After removing DCFDA, 100 μL/well of 1× Buffer were added and fluorescence was measured at Ex/Em = 485/535 nm. ROS production was reported as percentage of control.

### 4.4. Nuclear Protein Extraction

Nuclear proteins were extracted using the Nuclear Extraction Kit according to manufacturer’s instructions (Abcam plc, Cambridge, UK). Briefly, cell pellet (2 × 106 cells) was obtained by trypsinization and centrifugation of cells at 70–80% of confluence following standard protocols. Cell pellet was resuspended in 200 µL of pre-extraction buffer and incubated on ice for 10 min. After centrifugation, nuclear pellet was resuspended in 400 µL of extraction buffer and incubated on ice for 15 min. Finally, the suspension was centrifuged for 10 min at 14,000 rpm at 4 °C and the supernatant was transferred into a new vial to measure the protein concentration of the nuclear extract. Nuclear proteins quantification was performed by the Qubit Fluorometer (Invitrogen, Carlsbad, CA, USA) using the Qubit Protein Assay Kit according to manufacturer’s instructions.

### 4.5. DNMT Activity Quantification

Total DNMT activity was quantified using the colorimetric DNMT Activity Quantification Kit (Abcam plc, Cambridge, UK) according to manufacturer’s instructions. Briefly, 7.5 ng of nuclear extracts were diluted in 50 μL/well of reaction solution and incubated at 37 °C for 120 min, including blank and positive control. After removing the reaction solution, each well was rinsed with wash buffer for three times, and 50 μL/well of the diluted capture antibody was added. The plate was covered with aluminum foil and incubated at room temperature for 60 min. After removing the capture antibody, each well was rinsed with wash buffer for three times, and 50 μL/well of the diluted detection antibody was added. The plate was covered with aluminum foil and incubated at room temperature for 30 min. After removing the detection antibody, each well was rinsed with wash buffer for four times, and 50 μL/well of the enhancer solution was added. The plate was covered with aluminum foil and incubated at room temperature for 30 min. After removing the enhancer solution, each well was rinsed with wash buffer for five times, and 100 μL/well of the developer solution was added. Finally, the plate was covered with aluminum foil and incubated at room temperature for 10 min, away from direct light. When the positive control turned to medium blue, 100 μL/well of stop solution was added to stop the reaction. OD was read within 2–10 min at 450 nm with an optional reference wavelength of 655 nm. DNMT activity was reported as percentage of control.

### 4.6. SIRT1 Activity Quantification

SIRT1 activity was quantified using an SIRT1 Activity Assay Kit (Abcam plc, Cambridge, UK) according to manufacturer’s instructions. The reaction mixture containing 30 μL ddH_2_O, 5 μL fluorosubstrate peptide, 5 μL NAD, 5 μL developer, and 7.5 ng nuclear extract was mixed thoroughly, and the fluorescence intensity was measured at Ex/Em = 350–450 nm for 30 to 60 min at 1–2 min intervals. SIRT1 activity was reported as percentage of control.

### 4.7. Quantitative Real-Time Polymerase Chain Reaction (qPCR)

Total cellular RNA was extracted using Trizol^®^ Reagent (Invitrogen, Carlsbad, CA, USA) and reverse transcribed to single-stranded cDNA using the SuperScript III Reverse Transcriptase (Applied Biosystems, Foster City, CA, USA) according to the manufacturer’s protocols. mRNA levels were determined by qPCR with TaqMan Gene Expression Assays (Life Technologies, Monza, Italy) using the QuantStudio™ 7 Flex System (Applied Biosystems). Specific primers were used to detect DNMT1 (assay No. Hs00945875_m1), DNMT3a (Hs01027162_m1), DNMT3b (Hs00171876_m1), and SIRT1 (Hs01009006_m1). Data were normalized to the values of GAPDH expression (Hs02758991_g1). All samples were analyzed in triplicate using the 2^−ΔΔ*C*^_t_ method [57].

### 4.8. LINE-1 Methylation Analysis

DNA was extracted using the DNeasy Blood and Tissue Kit (Qiagen, Milan, Italy) and quantified using the Qubit dsDNA High Sensitivity Assay Kit (Life Technologies, Monza, Italy) according to the manufacturer’s protocols. Methylation analysis of three CpG sites in the LINE-1 promoter (GeneBank accession No. X58075) was performed by pyrosequencing of bisulfite-converted DNA using the PyroMark Q24 Instrument (Qiagen, Milan, Italy), as previously reported [58,59]. Briefly, 20 µg of DNA extracted from each sample was converted by bisulfite treatment using the Epitect Bisulfite Kit (Qiagen, Milan, Italy). Converted DNA was eluted in 20 μL of Elution buffer and stored at −80 °C until used. A reaction volume of 25 mL was amplified by polymerase chain reaction (PCR), using the PyroMark PCR Kit (Qiagen, Milan, Italy), according to the manufacturer’s instructions. Briefly, each reaction mixture contained 12.5 μL of PyroMark PCR Master Mix, 2.5 μL of CoralLoad Concentrate, 2 μL of the forward primer (5′-TTTTGAGTTAGGTGTGGGATATA-3′) and the reverse-biotinylated primer (5′-biotin-AAAATCAAAAAATTCCCTTTC-3′) (0.2 μM for each), and 1.5 μL of bisulfite-converted DNA. HotStart PCR cycling conditions were 1 cycle at 95 °C for 15 min, 40 cycles at 94 °C for 30 s, 50 °C for 30 s, 72 °C for 30 s, and a final extension at 72 °C for 10 min. The biotinylated PCR product was purified and made single-stranded using the Pyrosequencing Vacuum PrepTool (Biotage, Inc., Charlottesville, VA, USA). The biotinylated single-stranded product was annealed to the pyrosequencing primer (5′-AGTTAGGTGTGGGATATAGT-3′) and then subjected to sequencing using an automatically generated nucleotide dispensation order. The pyrogram was analyzed using allele quantification mode to determine the proportion of methylated and unmethylated cytosines. LINE-1 methylation level was reported as the average of the three specific CpG sites.

### 4.9. Statistical Analysis

Results were reported as MD ± SE unless otherwise indicated. Statistical differences in the data were evaluated by Student’s t-test using the control group and/or GOx- and LPS-treated cells as reference. All the analyses were conducted using GraphPad Version 6.0 with a significance level of 0.05.

## 5. Conclusions

In conclusion, we demonstrated for the first time that oxidative stress and inflammatory conditions negatively affect both DNMT and SIRT1 functions, and LINE-1 methylation in RPE cells. Interestingly, treatment with 10 μM resveratrol for 24 h counteracts these detrimental effects. Further studies should be encouraged to explore the perspectives of resveratrol as a suitable strategy for the prevention and/or treatment of retinal degenerative diseases.

## Abbreviations

AMD	Age-related macular degeneration
Aβ	Amyloid beta
ARPE-19	Human retinal pigment epithelial
CNV	Choroidal neovascularization
DCFDA	2′,7′–dichlorofluorescin diacetate
DMEM	Dulbecco’s Modified Eagle’s medium
DNMTs	DNA methyltransferases
GOx	Gluocose oxidase
GSTM1	Glutathione S-transferase isoforms mu1
GSTM5	Glutathione S-transferase isoforms mu5
IL17RC	Interleukin 17 receptor C
IL1β	Interleukin 1 beta
LINE-1	Long interspersed nuclear element-1
LPS	Lipopolysaccharid
MTT	Thiazolyl blue tetrazolium bromide
NF-κB	Nuclear factor-kappa B
qPCR	Quantitative real-time polymerase chain reaction
ROS	Reactive oxygen species
RPE	Retinal pigment epithelium
TNFα,	Tumor necrosis factor alpha
SIRT1	Sirtuin 1
